# How Can Transformative Sustainability Research Benefit From Integrating Insights From Psychology?

**DOI:** 10.3389/fpsyg.2021.676989

**Published:** 2021-06-17

**Authors:** Thomas Bruhn

**Affiliations:** Institute for Advanced Sustainability Studies (IASS), Potsdam, Germany

**Keywords:** transformative research, transdisciplinarity, relationality, mental model, leverage points

## Introduction—Essential Trends in Sustainability Research

Over the last decades, the field of sustainability science has experienced trends toward (1) a transdisciplinary (Hirsch Hadorn et al., 2006; Jahn et al., 2012) and (2) systemic, relationship-based understanding of transformation (Clark and Harley, 2020) and (3) transformative research (Schneidewind et al., 2016; Fazey et al., 2018; Clark and Harley, 2020). A key feature of these trends is that they emphasize the roles of human subjectivity and agency in transformation processes (Manuel-Navarrete, 2001, 2015; Lang et al., 2017). In the following I would like to briefly introduce these trends as basis for later discussion on how psychology could help address specific challenges in this context.

### From Environmental Science to Transdisciplinary Transformation Research

First, sustainability science has moved from focusing on the analysis of environmental issues toward a research field that aims at a transdisciplinary understanding of transformation (Kates, 2011). Discussions about sustainability were initially driven by environmental sciences and led to substantial research on resource efficiency, technological solutions, and their respective governance (Kates and Saito, 2001; Clark et al., 2005). Discussions on sufficiency and lifestyle changes originally attracted much less attention. Recently, this situation has shifted significantly. Many industrialized societies are facing challenges related to psychological health and well-being, stimulating the search for sustainable and mindful lifestyles (Kasser, 2003; Brown and Kasser, 2005). Today, there is broad agreement that human behavior patterns and lifestyles play crucial roles in the current crisis and influence future transformation pathways (Botkin et al., 2014; Lang et al., 2017). In light of this, integrating knowledge from various academic and non-academic sources has become a key feature of sustainability science.

### From “Top-Down vs. Bottom-Up” to a Systems-Based Theory of Change

Second, the discourse on sustainability has seen the emergence of new theories of change that are based particularly on an understanding of complex, adaptive systems (Clark and Harley, 2020), integrating insights from various research fields based on relational ontologies (Oberlack et al., 2019). Originally having an emphasis on environmental (i.e., Earth-system) changes, the discourse on sustainability used to have a certain bias toward “top-down” analyses and “solutions” to preserve the stability of global ecosystems (Lövbrand et al., 2009). In parallel, bottom-up activities have driven local change processes, leading, for example, to the transition movement and other initiatives. Only in the last decade, these perspectives have become increasingly integrated into what several authors call a “systems view” (Capra and Luisi, 2014) or “relational paradigm” (Walsh et al., 2020; West et al., 2020) of sustainability research and transformation.

### From Descriptive Science to Transformative Research

Third, the role of science in society has been shifting toward so-called transformative research that not only provides knowledge from a seemingly objective observer's point of view, but also actively engages with stakeholders to integrate academic understanding into processes of taking action (Lang et al., 2012). Not long ago, scientific discourse and organizations were largely focusing on research *about* sustainability phenomena, providing results as advice to decision-makers and preserving the “independence” of academia (Mobjörk, 2010). The boundaries of these roles have increasingly become blurred and scientists and research institutions are exploring how to contextualize research processes in multi-stakeholder processes that are normatively oriented toward the common good (Schneidewind et al., 2016; Fazey et al., 2018).

## Discussion—Why and How These Trends Call for an Integration of Psychology

All these trends are encountering challenges that create opportunities for psychology to contribute to sustainability-related research processes.

### Transdisciplinarity—On the Challenge of Overcoming Knowledge Hegemonies

In the context of developing a transdisciplinary understanding of transformation, psychology can contribute a lot of *knowledge* on how to integrate aspects of human behavior into transformation processes and how to understand the generation and representation of knowledge in transdisciplinary research processes.

A key issue of a transdisciplinary understanding of sustainability lies in the field of behavioral change and lifestyles. A lot of scientific advice for decision-makers is being provided based on so-called integrated assessment models (IAMs) that (implicitly or explicitly) include assumptions about collective behavior and behavioral change (van Vuuren et al., 2011; Béatrice et al., 2019). Undoubtedly, psychology can offer important insights and tools to understand the aspects and mechanisms shaping lifestyle choices and collective behavioral changes. Here, psychology scholars should be actively involved in the design of these models, e.g., to examine how the assumptions of these highly influential models are consistent with the latest psychological findings. For example, it seems crucial to me that sustainability-related discussions go beyond an individualistic understanding of the human being and its health and well-being. This could help create political incentive structures for behavior change that are not based on outdated understandings of the human being, like e.g., notions of a *homo economicus* which is still widespread in fields outside psychology, but widely criticized as inadequate in today's psychology and sociology literature (Urbina and Ruiz-Villaverde, 2019). While there have already been substantial efforts in the field of psychology to contribute to sustainability, I see a great need for sustainability-related research institutions and programs to integrate psychological perspectives more pro-actively into transdisciplinary research processes to account adequately for the role of human behavior.

Another important contribution could lie in helping to understand the factors that shape processes of effective knowledge integration (Wiek, 2007). Transdisciplinarity aims at integrating various forms of knowledge (i.e., systems knowledge, orientation knowledge, transformation knowledge and process knowledge). In practice, this includes non-academic knowledge and experiential or tacit knowledge, and many research processes are struggling with this ambition because they are lacking expertise on how to examine the factors that “lie behind” the ways different knowledge is being represented. Thus, it is highly relevant to understand the motivations, aspirations, and drivers that shape knowledge representations in these processes. Psychological perspectives can provide valuable expertise on how knowledge is generated and processed, for example through the integration of reflexive practices such as mindfulness in the research process (Lang et al., 2017).

### The Systems View—On the Challenge of Integrating Human Subjectivity

In the context of developing a systems-based theory of change, psychology can contribute a rich spectrum of empirical *methods* for investigating deeper systemic leverage points.

In a systems view, transformation processes are understood to be shaped by changing relationship patterns across systems and different leverage points for systemic change. Here, mental models, i.e., values, paradigms and belief systems, are considered as so-called deep-leverage points (Meadows, 1997; Abson et al., 2017).

Hence, as sustainability researchers are exploring the roles of subjectivity and mental models in transformation processes, they need methods that allow for an examination of these aspects. Psychology can either contribute its own, or help enhance existing non-psychological methods to integrate deeper and more complex understandings of human beings and their interactions in social contexts. As an example for this kind of synergetic work, I see the emerging community of so-called “psycho-social research” (Clarke, 2002, 2006; Clarke et al., 2018) that has integrated insights from psychoanalysis in the design of qualitative social science methods. Psycho-social research aims at reaching beyond narratives of a rational human being and tapping into the messy, contradictory, ambiguous “lived life,” e.g., by conducting life history interviews or by working with free associations and dreams (Hoggett, 2013).

Other exciting developments can be observed, for instance, in the context of adapting methods for systems constellations in contexts outside their origins in group or family psychology (Müller-Christ, 2018, 2019; Müller-Christ and Pijetlovic, 2018). Revealing patterns within human subjectivity and how they are reflected and manifested in inter-personal, social and even ecological relationships may play a key role in developing context-specific transformation strategies and practices. Researchers and organizations active in the context of sustainability should be open to the integration of these methods and the inclusion of related experts from psychology.

### Transformative Research—On the Challenge of Engaging Meaningfully

In the context of transformative research, I see that psychology has expertise in a broad range of *practice-oriented tools* that could contribute to integrating and improving reflexive elements for engaging stakeholders in research processes.

In transformative research, academics go beyond the notion of a seemingly independent scientific observer and actively engage with relevant stakeholders to co-design responses to present challenges. Specific challenges arise from the fact that the knowledge of the different stakeholders involved may be grounded in very different normative and ontological or epistemological assumptions. This means that the research process may only partly be about generating and evaluating knowledge. Rather, it may likely involve dynamics triggered, e.g., from interpersonal conflicts between different normative notions, values and worldviews or cultural and historical backgrounds. For handling such dynamics and conflicts, it is recommended to include reflexive or diffractive practices that invite all participants to reflect upon the normative implications of their own activities and examine their own subjective biases and how they might influence their notions and actions (Lang et al., 2017; Fazey et al., 2018).

Here, the insights and experiences from psychotherapy and psychodynamics can offer resources for designing formats of interaction and engagement. Often, I have experienced how transformative research processes became dysfunctional not because of lacking or inappropriate knowledge, but because of subtle (often implicit) power and oppression dynamics and subsequent emotional distress on the part of the participants. Sustainability researchers may be largely unaware of these dimensions of their work and scientific institutions often may not have the capacities to include professional facilitators that are trained to handle more profound conflicts and vulnerabilities. My experience is that sustainability-related conflicts—such as experience of injustice, colonialization, oppression, or marginalization—are influencing transformative research processes more than the responsible researchers are aware of. Fostering an understanding for the occurrence and careful handling of these dynamics seems crucial for successful transformative research in the future. Psychological schools have a successful history and solid evidence base to provide the expertise for addressing this gap.

As final outlook I would like to mention the idea of creating and holding specific spaces in which change agents can explore and transform their own behavior patterns and even institutional settings as part of transformation processes. For example, I have been very inspired by learning about the “carbon conversations” co-initiated by the psychotherapist Rosemary Randell in which citizens can collectively explore the psychological roots of and obstacles to their behavior and learn climate-friendly behavior patterns together (Randall, 2009). Also, in the context of organizational leadership, containment (Bion, 1985) is essential and well-established as a way to navigate change processes. I am wondering to what extent it might be possible to establish such spaces and routines of containment strategically for enabling transformation processes with stakeholder groups. It may seem a farfetched notion now, but in the face of the dawning ecological crises, the exhaustion and distress of the relevant stakeholders and institutions seems obvious to me, and it is becoming essential to open new pathways for working through existing conflicts. Psychological and psychotherapeutic approaches have gained significant expertise in how to design and conduct such processes to support personal health and well-being. For the sake of planetary health (Horton et al., 2014), maybe one day we will witness a kind of “*planetary containment initiative*.”

## Summary

I have reflected upon current trends in sustainability science and how psychology-based insights can contribute to addressing specific challenges arising as part of these trends. In the context of moving toward a transdisciplinary and systemic understanding of transformation and toward transformative research, psychology can contribute to a more holistic conceptualization of socio-ecological transformation. In particular, it can offer insights into the nature of human behavior and its interaction with social context dynamics Moreover, psychology can offer methods to describe patterns of human subjectivity and how they are entangled in larger systems dynamics. On the practical side, psychological practices can provide expertise on how to design and facilitate co-creative learning and meaning-making spaces that go beyond creative practice: by allowing for the exploration and transformation of deeper root causes of conflicts that are often inherent to stakeholder engagement.

## Author Contributions

TB has conceptualized and written the manuscript.

## Conflict of Interest

The author declares that the research was conducted in the absence of any commercial or financial relationships that could be construed as a potential conflict of interest.
